# Supplementary material for: The prevalence of diabetes amongst young Emirati female adults in the United Arab Emirates: A cross-sectional study
Source: PLoS One. 2021 Jun 17;16(6):e0252884. doi: 10.1371/journal.pone.0252884 (PMC8211155; doi:10.1371/journal.pone.0252884)
Supplement: Attachment — Submitted filename: PONE-D-20-38524_reviewer Coments .pdf [file pone.0252884.s001.pdf]

PLOS ONE

Manuscript No. PONE-D-20-38524 22nd February 2021

Dear Editor Muhammad Sajid Hamid Akash, PhD

TITLE: The prevalence of diabetes amongst young Emirati female adults in the United Arab Emirates: A cross-sectional study

Author: Maysm Nezar Mohamad et al.

Comments to the Author

This manuscript nicely presented, an interesting finding that the cross-sectional study of 555 female college students aged 17-25, enrolled at United Arab Emirates University in Al Ain, UAE. The Anthropometric analysis, blood pressure, and various biochemical markers were measured using standard methods. Type 2 Diabetes, impaired fasting plasma glucose (FPG), and elevated HbA1c levels were examined in the study. Overall, these findings are important and interesting. However, further, improvement is necessary to solidify the Manuscript.

Here are a few comments and questions:

1. When this study was conducted? Please rationalize the selection of target population. Why the aged 17– 25 students are selected?
2. How you elaborated the other marker like blood pressure with diabetes or pre-diabetic cases?
3. Why only university students were focused, please add in the study limitation and justify the sampling biased.
4. What is the major objective of the study? Please focus on it. If its correlation, please explain it well.
5. Correction needed in writing "SPSS software, version 26.0 (SPSS, Chicago, IL, UASA)".
6. Explain about each test where Univariate and where multivariate logistic regressions was applied.

Authors address these deficiencies, then the manuscript should be considered for publication.
